# What is the role of clinical tests and ultrasound in acetabular labral tear diagnostics?

**DOI:** 10.3109/17453670902988402

**Published:** 2009-06-01

**Authors:** Anders Troelsen, Inger Mechlenburg, John Gelineck, Lars Bolvig, Steffen Jacobsen, Kjeld Søballe

**Affiliations:** ^1^Orthopaedic Research Unit, Aarhus University HospitalCopenhagenDenmark; ^2^Department of Radiology, Aarhus University HospitalAarhusDenmark; ^3^Department of Orthopaedic Surgery, Hvidovre University HospitalCopenhagenDenmark

## Abstract

**Background and purpose** An acetabular labral tear is a diagnostic challenge. Various clinical tests have been described, but little is known about their diagnostic sensitivity and specificity. We investigated the diagnostic validity of clinical tests and ultra-sound as compared with MR arthrography.

**Patients and methods** We examined 18 patients (18 hips, 2 men, median age 43 (32–56) years) with impingement test, FABER test, resisted straight leg raise test, ultrasound, and MR arthrography. They had had previous periacetabular osteotomies due to symptomatic, acetabular dysplasia. All hips showed no or only slight signs of osteoarthritis (Tönnis grade 0–1).

**Results** MR arthrography identified labral tears in 17 of the 18 hips. Ultrasound had a sensitivity of 94%, a positive predictive value of 94%, and was false negative in only 1 case compared to MR arthrography. The impingement test had the best diagnostic ability of the clinical tests, with a sensitivity of 59% and a specificity of 100%. The positive predictive value was 100% while the negative predictive value was 13%.

**Interpretation** The impingement test is helpful in identifying acetabular labral tears. If this test is negative and if a labral tear is still suspected, ultrasound can reliably diagnose most tears of the acetabular labrum. MR arthrography is indicated in cases where ultrasound is negative, but the patient suffers continued, specific symptoms.

## Introduction

Acetabular labral tearing is frequently encountered in patients with acetabular dysplasia or femoroacetabular impingement, and remains a diagnostic challenge (Byrd and Jones 2003, Wenger et al. 2004). MR arthrography has been the diagnostic gold standard (Chan et al. 2005, Freedman et al. 2006, Toomayan et al. 2006). An investigation of ultrasound has shown poor diagnostic value (Troelsen et al. 2007). Various clinical tests for diagnosing acetabular labral tears are used: the impingement test, the FABER test, and the resisted straight leg raise test (Kelly et al. 2003, Martin et al. 2006). Yet, little is known about their sensitivity and specificity (Mitchell et al. 2003, Narvani et al. 2003). We therefore investigated the diagnostic value of clinical tests and ultrasound and compared it with that of MR arthrography.

## Patients and methods

Clinical examinations, ultrasound, and MR arthrography were performed between December 2006 and June 2007. The study was approved by the regional scientific ethics committee (no. 2003 0021) and informed consent was obtained from all patients. 30 patients who had had periacetabular osteotomies between April 2003 and June 2004 (30 hips) and who visited our institution to participate in another scientific investigation were eligible for inclusion. 1 patient was excluded due to conversion to total hip arthroplasty, 1 had emigrated, 2 were non-responders, 7 did not wish to participate, and 1 who did not show up for MR arthrography was excluded after the study was started. Thus, the study group consisted of 18 patients (18 hips, 2 men). Median age was 43 (32–56) years. Patients had developmental dysplasia of the hip with preoperative center-edge angles of < 25˚ (Wiberg 1939). All hip joints had no or only slight signs of radiographic degeneration (Tönnis grade 0–1) preoperatively and at the latest follow-up (Tönnis 1987). After periacetabular osteotomies, the median center-edge angle was 35° (29–40).

MR arthrographies were performed by one senior radiologist, and intraobserver variability of MR arthrograms was assessed by doing masked re-readings separated by 4 weeks, ultrasound by one senior radiologist, and clinical tests by a specially instructed orthopedic resident—all without knowing the findings of the others.

### Clinical testing

**Figure 1.**
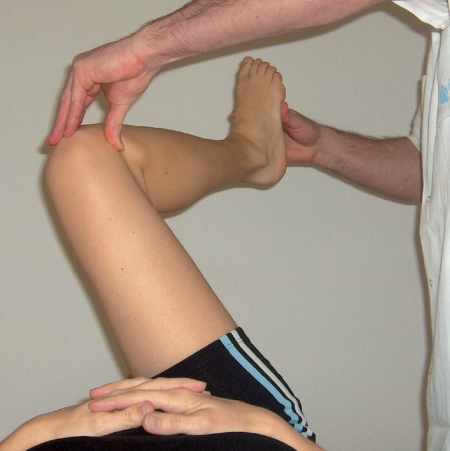
The impingement test was carried out by passively moving the hip joint in flexion (to 90°), internal rotation, and adduction. The test was regarded as being positive on reproduction of groin pain.

**Figure 2.**
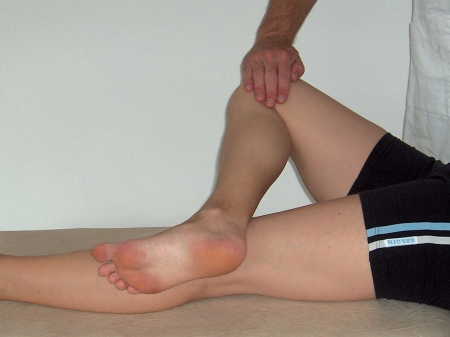
In the FABER (Flexion, ABduction, External Rotation) test, the hip joint was passively flexed, abducted, and externally rotated with the knee flexed (figure-of-four-position). The ankle was brought to rest just above the contralateral knee and slight pressure was applied to the medial side of the knee, approximating it to the examination table. The test was regarded as being positive on reproduction of groin pain.

**Figure 3.**
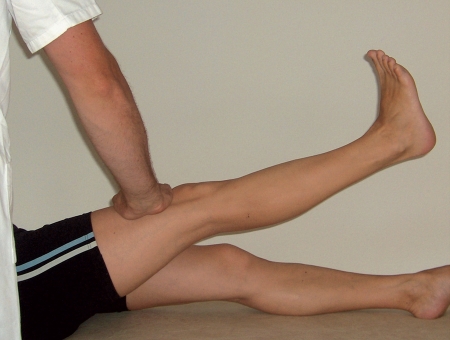
The resisted straight leg raise test. The hip joint was actively flexed to approximately 30° with the knee extended. This position was held by the patient while the examiner applied pressure on the extremity just above the knee, toward the examination table. The test was regarded as being positive on reproduction of groin pain.

In the impingement test the hip joint was passively flexed to 90˚, internally rotated, and adducted (Figure 1). In the FABER test the lower extremity was passively placed in a figure-of-four position, and slight pressure was applied to the medial side of the knee (Figure 2). In the resisted straight leg raise test, the patient actively flexed the hip joint to approximately 30˚ with extended knee. This position was held while the examiner applied a downward pressure (Figure 3). The patient’s response was registered as follows: (1) no pain; (2) groin pain; (3) anterior thigh pain; (4) posterior thigh pain; (5) lateral thigh pain; (6) pain in the buttock; (7) knee pain; (8) lumbarsacral or sacral-iliac pain; and (9) pain in other regions. A test was regarded positive by reproduction of groin pain.

### Ultrasound examination

Ultrasound examinations were performed using a Hitachi model EUB-8500 (Hitatchi Medical Corporation, Tokyo, Japan) and a 13–MHz (13-6 MHz) linear transducer resolution of 0.1–0.2 mm). The transducer was initially placed parallel to the femoral neck, with identification of the femoral head and neck. It was then moved across the hip joint and tilted for evaluation of the labrum in the lateral acetabular quadrants. In addition, the labrum was visualized as the hip was moved passively in flexion, adduction and internal rotation. This dynamic examination was used to reveal labral detachment. The ultra-sound examination took 5–10 minutes. Criteria for labral tears were: (1) displacement or (2) absence of the labrum; (3) hypoechoic cleft through the base of the labrum causing detachment with or without displacement; (4) intra-substance hypoechoic linear clefts; intra-substance hypoechoic (5) cystic or (6) irregular formations. Mixed echogenicity without definite tearing and irregular margins was interpreted as degenerative changes (Troelsen et al. 2007)

### MR arthrography

Guided by fluoroscopy, 8 mL of diluted gadolinium contrast medium (Gd-DTPA, 2 mmol/L; Magnevist; Bayer AG, Berlin, Germany) was injected into the hip joint. Before injection, the intraarticular position of the needle point was verified by injecting a few drops of iodinated contrast medium. There were no adverse effects. MR arthrographies were performed with a 1.5 Tesla Scanner (Siemens Magnetom Symphony; Siemens, Erlangen, Germany). Initially, 3 scout sequences in the axial, sagittal, and coronal planes were obtained, followed by T1-weighted sequences with fat suppression: true coronal, oblique axial (parallel to the femoral neck), oblique coronal 45˚ forward-angled, and oblique coronal 45˚ backward-angled (TR/TE 376/20, slice thickness 4 mm, field of view 220 × 220, and matrix 256 × 256). Finally, a coronal STIR sequence through the entire pelvis was performed (TR/TE 410/27, TI 170, field of view 400 × 400, and matrix 256 × 256). Criteria for labral tears were: (1) displacement or (2) absence of the labrum; (3) contrast medium through the base of the labrum causing detachment with or without displacement; intra-substance (4) linear, (5) cystic, or (6) irregular presence of contrast medium. Intermediate signal intensity and irregular margins were interpreted as degenerative changes (Troelsen et al. 2007).

In all periacetabular osteotomies, the acetabular fragment had been fixed using long cortical screws made of titanium. The titanium screws caused only minimal artefacts in the MR arthrograms, and full diagnostic assessment was not compromised in any of the cases.

### Statistics

Sensitivity, specificity, positive predictive value, and negative predictive value were calculated to describe the value of clinical tests and ultrasound in acetabular labral tear diagnostics.

## Results

**Figure 4.**
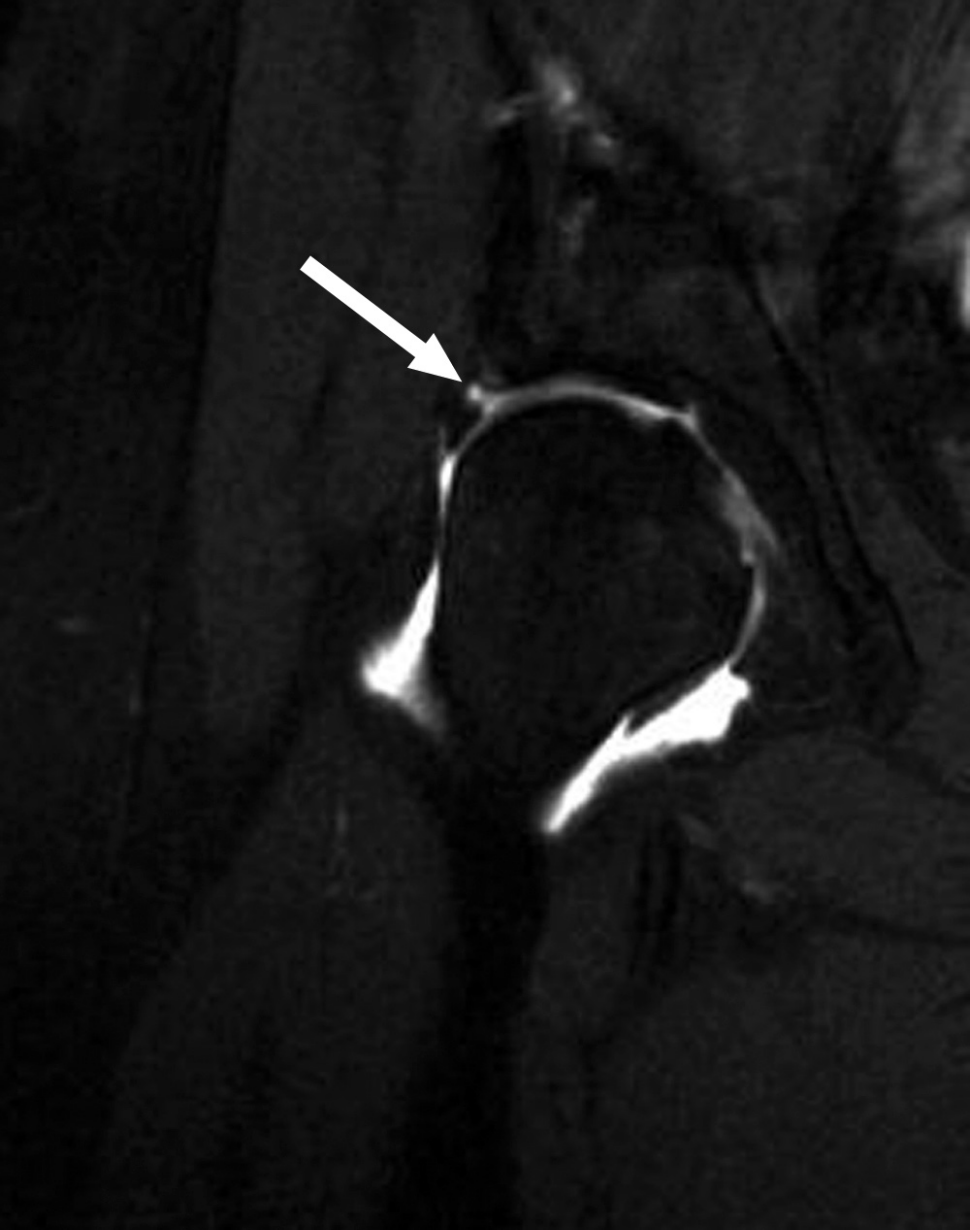
An acetabular labral tear visualized by MR arthrography (coronal view) with contrast medium running through the base of the labrum.

Acetabular labral tears were identified in 17 of the 18 hip joints on MR arthrograms (Figure 4). The agreement observed between blinded re-readings of MR arthrograms was 100%.

**Figure 5.**
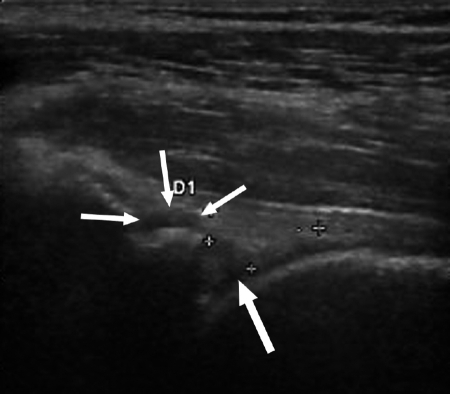
An acetabular labral tear visualized with ultrasound. The crosses mark the approximate limits of the triangular-shaped labrum. The thick arrow points to the hypoechoic cleft that runs through the base of the labrum, ending in cystic formation just superior to the labrum (3 thin arrows).

Ultrasound visualized labral tears in 17 of 18 hip joints (Figure 5). Of these, 16 were confirmed by MR arthrography, whereas 1 was false-positive and another was found to be false-negative (Table 1). Thus, ultrasound in labral tear diagnostics had a sensitivity of 94% and a positive predictive value of 94%. We found no true negatives, i.e. absence of a labral tear in both ultrasound and MR arthrography. Specificity and negative predictive value were therefore not assessed (Table 4).

**Table 1.** Comparison of findings of MR arthrography and ultrasound

	Ultrasound		
MR-arthrography	Labral tear	No labral tear	Total
Labral tear	16	1	17
No labral tear	1	0	1
Total	17	1	18

The impingement test was positive in 10 of 18 cases. In these cases, MR arthrography confirmed the presence of labral tears (i.e. true positives). In 7 cases the test was false negative and in 1 case it was true negative (Table 2). Accordingly, the impingement test had a sensitivity of 59% and a specificity of 100%. The positive predictive value was 100%, and the negative predictive value was 13% (Table 4).

**Table 2.** Comparison of findings of MR arthrography and impingement test

	Impingement test		
MR-arthrography	Positive	Negative	Total
Labral tear	10	7	17
No labral tear	0	1	1
Total	10	8	18

The FABER test was positive in 7 of 18 cases, all of which were confirmed as true positives. The test was false negative in 10 cases and true negative in one case (Table 3). Thus, the sensitivity was 41% and the specificity was 100%. The positive predictive value was 100% and the negative predictive value was 9% (Table 4).

**Table 3.** Comparison of findings of MR arthrography and FABER test

	FABER test		
MR-arthrography	Positive	Negative	Total
Labral tear	7	10	17
No labral tear	0	1	1
Total	7	11	18

The resisted straight-leg raise test was positive in only 1 of 18 cases. In this case, the other clinical tests were also positive, and both ultrasound and MR arthrography confirmed the presence of a labral tear.

## Discussion

Using MR arthrography as diagnostic gold standard, we found that clinical tests are at best potentially helpful and that ultra-sound examination is highly reliable in acetabular labral tear diagnostics.

Findings of MR arthrography were not verified by hip arthroscopy. Comparison of alternative examining modalities to MR arthrography is appropriate since previous studies have reported very high sensitivity, specificity, and accuracy of MR arthrographic findings compared to those of hip arthroscopy (Chan et al. 2005, Freedman et al. 2006, Toomayan et al. 2006). Assessment of intra- and interobserver variability for ultra-sound examinations would have given interesting information. However, ultrasound examinations of the acetabular labrum demand experienced examiners and due to logistic obstacles, intra- and interobserver variability were not assessed. Furthermore, the study was limited by the frequent presence of acetabular labral tears in a selected group, making assessment of the diagnostic ability in cases of a normal labrum difficult. However, this setting reflects the clinical norm in outpatient clinics dealing specifically with hip-related problems. Examinations were carried out prospectively and the examiners were blinded as to the findings of the others, which gave extra methodological strength.

Few authors have investigated the role of ultrasound in ace-tabular labral tear diagnostics (Mitchell et al. 2003, Sofka et al. 2006, Troelsen et al. 2007). One study reported a sensitivity of 44%, a specificity of 75%, a positive predictive value of 88%, and a negative predictive value of 25% (Troelsen et al. 2007). From that study, it was concluded that the potential of ultrasound in this specific context should be developed further before definite recommendations could be made. The two remaining studies reported a sensitivity of 13% and a specificity of 100% (Mitchell et al. 2003), and a subjective improvement in visualization of labral morphology following intraarticular steroid injections (Sofka et al. 2006). In the present study, ultrasound was highly reliable in labral tear diagnostics when used in experienced hands (Table 4). The clearly improved diagnostic ability compared to the previous study (Troelsen et al. 2007) indicates that a learning curve may be associated with the use of ultrasound in acetabular labral tear diagnostics, in this case even for the experienced examiner who did all ultrasound examinations in both studies. However, the ability of ultrasound to diagnose acetabular labral tears in an unselected group of patients with hip-related problems remains unclear.

**Table 4.** Diagnostic value of ultrasound and clinical tests compared to MR arthrogra-phy in labral tear diagnostics

	Ultrasound	Impingement test	FABER test
Sensitivity	16/17 = 94%	10/17 = 59%	7/17 = 41%
Specificity	0/1 = 0%	1/1 = 100%	1/1 = 100%
Positive predictive value	16/17 = 94%	10/10 = 100%	7/7 = 100%
Negative predictive value	0/1 = 0%	1/8 = 13%	1/11 = 9%

Very little knowledge has accumulated regarding the diagnostic ability of the impingement test, the FABER test, and the resisted straight leg raise test in the specific diagnosis of ace-tabular labral tears. In a study on 18 hips using MR arthrography as reference, the sensitivity and specificity of an “internal rotation, flexion, axial compression” manoeuvre were reported to be 75% and 43% (Narvani et al. 2003). The study reported 8 cases of false-positive outcomes, giving a positive predictive value of 27%. In 3 studies reporting clinical findings in patients with surgically verified labral tears and with the impingement test carried out in a similar way to that in our study, the impingement test was positive in ≥ 95% of cases (Fitzgerald 1995, Ito et al. 2004, Burnett et al. 2006). In the present study, the impingement test showed the best ability to diagnose acetabular labral tears (Table 4). If groin pain can be reproduced by the impingement or FABER tests, the patient is very likely to have a labral tear. However, a negative outcome for the impingement test or the FABER test is unreliable and further radiographic assessment will be needed. Given the discrepancy between the number of acetabular labral tears diagnosed on MR arthrograms and the number of positive impingement and FABER tests, one could ask if there might be a difference between labral tears that produce positive and negative outcomes in clinical tests. The present study does not support any conclusions on this matter. As for ultrasound, the diagnostic ability of clinical tests in an unselected group of patients with hip-related problems remains unclear.
